# Qualidade de Vida Relacionada à Saúde e Resultados em Longo Prazo após Covid-19 Levemente Sintomático: Explorando o Protocolo

**DOI:** 10.36660/abc.20230639

**Published:** 2023-10-06

**Authors:** Lucas Borges Pereira, João Paulo Vilela Rodrigues, Fabiana Rossi Varallo, Leonardo Régis Leira Pereira

**Affiliations:** 1 Faculdade de Ciências Farmacêuticas de Ribeirão Preto Universidade de São Paulo Ribeirão Preto SP Brasil Faculdade de Ciências Farmacêuticas de Ribeirão Preto , Universidade de São Paulo , Ribeirão Preto , SP - Brasil; 2 Faculdade de Ciências da Saúde de Barretos Dr. Paulo Prata Barretos SP Brasil Faculdade de Ciências da Saúde de Barretos Dr. Paulo Prata , Barretos , SP - Brasil

**Keywords:** Covid-19, SARS-CoV-2, Inflamação/complicações, Miocardite, Infarto do Miocárdio, Arritmias Cardíacas, Tromboembolia

A Síndrome Pós-Covid-19 foi definida pela Organização Mundial da Saúde como uma “condição que ocorre em indivíduos com histórico de infecção provável ou confirmada por SARS-CoV-2, normalmente após três meses do início da Covid-19, com sintomas que duram pelo menos dois meses e que um diagnóstico alternativo não pode explicar”. Os principais sintomas associados a esta condição incluem fadiga, dores musculares e articulares, falta de ar, dificuldades de sono, sintomas mentais e neurológicos como depressão e ansiedade, perda de olfato e paladar, dor de cabeça e dificuldade de pensamento e concentração. ^1 , 2^ O efeito citopático do vírus no endotélio, juntamente com o dano endotelial como consequência da inflamação sistêmica, tem sido associado a eventos cardiovasculares como miocardite, infarto do miocárdio, arritmias, e eventos tromboembólicos. ^3^

Vários estudos realizados em todo o mundo também encontraram evidências de outros sinais e sintomas. ^4 - 7^ Entretanto, a incidência desses sintomas tem apresentado variação significativa devido à heterogeneidade dos estudos. Essa variação pode estar associada a fatores como o período de estudo pós-COVID, a região onde o estudo foi realizado e a gravidade da doença. ^4^

Estima-se que até 2021, 3,92 bilhões de pessoas tenham sido infectadas pelo vírus SARS-CoV-2, e 3,7% tenham desenvolvido a condição pós-Covid-19. ^1^ Devido ao elevado número de pessoas que já foram infectadas pelo menos uma vez e as evidências limitadas sobre as consequências que esta doença pode ter nos pacientes, estudos prospectivos em indivíduos infectados pelo SARS-CoV-2 podem contribuir para uma melhor compreensão desta condição, especialmente a longo prazo.

O manuscrito “ Qualidade de Vida Relacionada à Saúde e Desfechos em Longo Prazo após Covid-19 Sintomática Leve: Protocolo do Estudo Pós-Covid Brasil 2” tem como objetivo avaliar fatores associados à qualidade de vida dos pacientes um ano após infecção leve por SARS-CoV-2 no Brasil. Os autores também propõem estudar os períodos de 3, 6 e 9 meses após a infecção. ^8^

O Brasil é um país que oferece um excelente campo de estudo para esse problema de saúde, pois possui uma das maiores incidências de Covid-19 no mundo. ^9^ Os estudos brasileiros abrangeram amostras regionalizadas ^10 - 13^ e estudos longitudinais com observação de curto período ou desenhos transversais, ^11 , 14^ Rover et al. pretendem analisar a população brasileira além do período de estudo de um ano. ^8^ Porém, não serão incluídos centros da região Nordeste, e apenas um centro da região Centro-Oeste e um centro da região Norte serão incluídos, colocando-se assim uma limitação ao estudo. Vale ressaltar que a maior incidência de Covid-19 no Brasil ocorre nas regiões Sul e Centro-Oeste. ^9^

Outro ponto forte deste estudo é a quantidade de informações que serão coletadas para compreender as manifestações das condições pós-Covid-19. Qualidade de vida, mortalidade, complicações, sintomas, estado funcional, dados demográficos, comorbidades, situação vacinal e exames laboratoriais serão obtidos para esta avaliação. Muitas informações serão obtidas por meio de entrevistas telefônicas, uma estratégia apropriada para um estudo multicêntrico. No entanto, os autores não informaram uma duração estimada da entrevista, e sabe-se que entrevistas longas apresentam maior risco de coleta de dados errôneos devido ao cansaço do entrevistado e do entrevistador.

Apesar das limitações, acreditamos que o estudo seja relevante, uma vez que os trabalhos publicados sobre condições pós-Covid-19 se concentraram principalmente na progressão de pacientes com história de infecção moderada a grave. Portanto, os dados sobre os resultados em longo prazo de pacientes que tiveram doença leve que incluíram indivíduos de diferentes regiões brasileiras são escassos. Além disso, os resultados deste estudo irão melhorar o trabalho dos profissionais de saúde no cuidado de pacientes infectados pelo SARS-CoV-2.
