# Supplementary material for: Qualidade de Vida Relacionada à Saúde e Resultados em Longo Prazo após Covid-19 Levemente Sintomático: Explorando o Protocolo
Source: Arq Bras Cardiol. 2023 Oct 6;120(9):e20230639. [Article in Portuguese] doi: 10.36660/abc.20230639 (PMC10567079; doi:10.36660/abc.20230639)
Supplement: Translation

# Translation: Health-related Quality of Life and Long-term Outcomes after Mildly Symptomatic COVID-19: Exploring the Protocol

**Authors:** Lucas Borges Pereira, João Paulo Vilela Rodrigues, Fabiana Rossi Varallo, Leonardo Régis Leira Pereira

**Affiliations:** 1 Brasil Faculdade de Ciências Farmacêuticas de Ribeirão Preto, Universidade de São Paulo, Ribeirão Preto, SP - Brasil; 2 Brasil Faculdade de Ciências da Saúde de Barretos Dr. Paulo Prata, Barretos, SP - Brasil

**Keywords:** COVID-19, SARS CoV-2, Inflammation/complications, Myocarditis, Myocardial Infarction, arrhythmias Cardiac, Thromboembolism

The post-COVID-19 condition has been defined by the World Health Organization as a “condition that occurs in individuals with a history of probable or confirmed SARS-CoV-2 infection, typically after three months from the onset of COVID-19, with symptoms lasting at least two months and that an alternative diagnosis can not explain.” The primary symptoms associated with this condition include fatigue, muscle and joint pain, shortness of breath, sleep difficulties, mental and neurological symptoms such as depression and anxiety, loss of smell and taste, headache, and difficulty in thinking and concentrating. ^1 , 2^ The cytopathic effect of the virus on the endothelium, along with endothelial damage as a consequence of systemic inflammation, has been associated with cardiovascular events such as myocarditis, myocardial infarction, arrhythmias, and thromboembolic events. ^3^

Various studies conducted worldwide have also found evidence of other signs and symptoms. ^4 - 7^ However, the incidence of these symptoms has shown significant variation due to the heterogeneity of the studies. This variation may be associated with factors such as the post-COVID study period, the region where the study was conducted, and the severity of the disease. ^4^

It is estimated that by 2021, 3.92 billion people had been infected with the SARS-CoV-2 virus, and 3.7% had developed the post-COVID-19 condition. ^1^ Due to the high number of people who have already been infected at least once and the limited evidence regarding the consequences this disease can have on patients, prospective studies in individuals infected with SARS-CoV-2 can contribute to a better understanding of this condition, especially in the long term.

The manuscript “Health-related quality of life and long-term outcomes after mildly symptomatic COVID-19: The post-COVID Brazil study protocol” aims to evaluate factors associated with patients’ quality of life one year after mild SARS-CoV-2 infection in Brazil. The authors also propose to study the periods of 3, 6, and 9 months after infection. ^8^

Brazil is a country that provides an excellent field of study for this health issue, as it has one of the highest incidences of COVID-19 in the world. ^9^ Brazilian studies have covered regionalized samples ^10 - 13^ and longitudinal studies with a short period observation or cross-sectional designs. ^11 , 14^ Rover et al. intend to analyze the Brazilian population in addition to the one-year study period. ^8^ However, centers from the Northeast region will not be included, and only one center from the Central-West and one from the North regions will be included, thus posing a limitation to the study. It is worth noting that the highest incidence of COVID-19 in Brazil occurs in the South and Central-West regions. ^9^

Another strength of this study is the amount of information that will be collected to understand the manifestations of post-COVID-19 conditions. Quality of life, mortality, complications, symptoms, functional status, demographic data, comorbidities, vaccination status, and laboratory tests will be obtained for this assessment. Much information will be obtained through telephone interviews, an appropriate strategy for a multi-center study. However, the authors did not report an estimated duration of the interview, and it is known that long interviews carry a higher risk of collecting erroneous data due to interviewee and interviewer fatigue.

Despite the limitations, we believe the study is relevant since the published works on post-COVID-19 conditions have mainly focused on the progression of patients with a history of moderate to severe infection. Therefore, data on the long-term outcomes of patients who had mild disease that included individuals from different Brazilian regions are scarce. Furthermore, this study’s results will enhance healthcare professionals’ work in caring for patients infected with SARS-CoV-2.
